# Return to work after subacromial decompression, diagnostic arthroscopy, or exercise therapy for shoulder impingement: a randomised, placebo-surgery controlled FIMPACT clinical trial with five-year follow-up

**DOI:** 10.1186/s12891-021-04768-7

**Published:** 2021-10-19

**Authors:** Mathias Bäck, Mika Paavola, Pasi Aronen, Teppo L. N. Järvinen, Simo Taimela, for the Finnish Shoulder Impingement Arthroscopy Controlled Trial (FIMPACT) Investigators

**Affiliations:** 1 Finnish Centre for Evidence-Based Orthopaedics (FICEBO), Department of Orthopaedics and Traumatology, University of Helsinki and Helsinki University Hospital, Töölö Hospital, P.O. Box 266, 00029 HUS, Helsinki, Finland; 2 Department of Orthopaedics and Traumatology, Helsinki University Hospital, Helsinki, Finland; 3 Biostatistics Unit, Faculty of Medicine in the University of Helsinki and Helsinki University Hospital, Helsinki, Finland

**Keywords:** Subacromial pain, Randomised controlled trial, Subacromial decompression, Placebo surgery, Exercise therapy, Return to work

## Abstract

**Background:**

Arthroscopic subacromial decompression is one of the most commonly performed shoulder surgeries in the world. It is performed to treat patients with suspected shoulder impingement syndrome, i.e., subacromial pain syndrome. Only few studies have specifically assessed return-to-work rates after subacromial decompression surgery. All existing evidence comes from open, unblinded study designs and this lack of blinding introduces the potential for bias. We assessed return to work and its predictors in patients with shoulder impingement syndrome in a secondary analysis of a placebo-surgery controlled trial.

**Methods:**

One hundred eighty-four patients in a randomised trial had undergone arthroscopic subacromial decompression (*n* = 57), diagnostic arthroscopy, a placebo surgical intervention, (*n* = 59), or exercise therapy (*n* = 68). We assessed return to work, defined as having returned to work for at least two follow-up visits by the primary 24-month time point, work status at 24 and 60 months, and trajectories of return to work per follow-up time point. Patients and outcome assessors were blinded to the assignment regarding the arthroscopic subacromial decompression vs. diagnostic arthroscopy comparison. We assessed the treatment effect on the full analysis set as the difference between the groups in return-to-work rates and work status at 24 months and at 60 months using Chi-square test and the predictors of return to work with logistic regression analysis.

**Results:**

There was no difference in the trajectories of return to work between the study groups. By 24 months, 50 of 57 patients (88%) had returned to work in the arthroscopic subacromial decompression group, while the respective figures were 52 of 59 (88%) in the diagnostic arthroscopy group and 61 of 68 (90%) in the exercise therapy group. No clinically relevant predictors of return to work were found. The proportion of patients at work was 80% (147/184) at 24 months and 73% (124/184) at 60 months, with no difference between the treatment groups (*p*-values 0.842 and 0.943, respectively).

**Conclusions:**

Arthroscopic subacromial decompression provided no benefit over diagnostic arthroscopy or exercise therapy on return to work in patients with shoulder impingement syndrome. We did not find clinically relevant predictors of return to work either.

**Trial registration:**

ClinicalTrials.gov identifier NCT00428870.

## Background

Subacromial pain, shoulder impingement syndrome and rotator cuff disease describe the most common shoulder conditions. The syndrome is distinctively characterised by pain located subacromially while lifting the arm and has traditionally been attributed to ‘impingement’ of the rotator cuff tendons between the humeral head and the overlying acromion. Following to this logic, a surgical procedure called subacromial decompression was developed. It is a surgical procedure premised on smoothening the undersurface of the acromion, which decompresses the rotator cuff tendons passing through the subacromial space. With the advent of arthroscopy, arthroscopic subacromial decompression (ASD) became a safe, quick and convenient procedure, and thus became one of the most frequently performed orthopaedic surgical procedures in the world [1].

A Cochrane review [2] and a BMJ Rapid Recommendation [3], two recently published systematic analyses of the evidence [4, 5], summarized that ASD offers no clinically relevant benefits on shoulder symptoms and function over nonoperative treatment or placebo surgery. However, some advocates of the surgery and some authorities (funders, insurance companies) still defend the appropriateness of the procedure based on its perceived ability to enable people to return to work more quickly. Evidence from previous trials on ASD (vs. non-operative treatment, usually exercise therapy) have reported rates of return to work (RTW) that support such contention [6, 7], and other trials do not support it [5, 8, 9]. Given the unblinded, open design of all these previous studies, the data may be prone to bias. Accordingly, we conducted a secondary analysis of our data from our recently published placebo-surgery controlled FIMPACT trial [5, 10, 11], where we compared the rates of RTW in patients who had undergone arthroscopic subacromial decompression (ASD), diagnostic arthroscopy (DA, a placebo surgical intervention), and exercise therapy (ET, a non-operative alternative) among patients with shoulder impingement syndrome. We also assessed whether age, gender, baseline work status, and strain at work were potential predictors of RTW.

## Methods

### Trial design and setting

We conducted this superiority trial at three orthopaedic clinics (Hatanpää hospital, Tampere, Pirkanmaa Hospital District; Jorvi and Herttoniemi hospitals, Helsinki, Helsinki University Hospital District) in Finland from February 1, 2005 through October 10, 2018. The study was registered at ClinicalTrials.gov (NCT00428870, on 30/01/2007). Details of the trial design and conduct have been published elsewhere [5, 10]. The patients and those who collected the data regarding the ASD vs. DA comparison were blinded to the study-group assignments. The protocol was approved by the Institutional Review Board of the Pirkanmaa Hospital District (R04200).

The study was conducted in accordance with the Declaration of Helsinki. All patients gave written informed consent. On entering the study, they were unequivocally informed that they might undergo diagnostic arthroscopy and that they would be allowed to consider crossing over to ASD if they did not have adequate relief of symptoms, preferably no sooner than 6 months after randomisation.

### Participants

We enrolled patients 35 to 65 years old who had subacromial pain (for more than 3 months) that was unresponsive to conventional conservative treatment and had clinical findings consistent with shoulder impingement syndrome. Magnetic resonance imaging with intra-articular contrast was performed on all patients to exclude a rotator cuff tear. Detailed inclusion and exclusion criteria are provided in Table 1.

**Table 1:** Inclusion and exclusion criteria

**Inclusion criteria**	
1. Adult men or woman ages 35 to 65 years.	
2. Subacromial pain for greater than 3 months with no relief from non-operative means (physiotherapy, non-steroidal anti-inflammatory medication, corticosteroid injections, and rest).	
3. Pain provoked by abduction and positive painful arc -sign.	
4. Positive impingement test (temporary relief of pain by subacromial injection of lidocaine).	
5. Pain in at least 2 out of 3 isometric tests (abduction 0° and 30° or external rotation).	
6. Provision of informed consent from the patient. 7. Ability to speak, understand and read in the language of the clinical site.	
**Exclusion criteria**	
1. Full thickness tear of the rotator cuff tendons diagnosed on clinical examination (marked weakness in any of the examined muscles) or magnetic resonance imaging with intra-articular contrast (MRA).	
2. Osteoarthritis of the glenohumeral and/or acromioclavicular joint diagnosed on clinical examination or on x-rays.	
3. Substantial calcific deposits in the rotator cuff tendons found in the preoperative imaging.	
4. Previous surgical procedure on the affected shoulder.	
5. Evidence of the shoulder instability (positive apprehension/positive sulcus sign).	
6. Symptomatic cervical spine pathology.	
7. History of alcoholism, drug abuse, psychological or psychiatric problems that are likely to invalidate informed consent.	
8. Patient declined to participate.	

### Randomisation and blinding

In an attempt to obtain three balanced study groups of similar group size, we planned a two-fold, sequential randomization as follows: First, during the baseline appointment, patients were randomized to surgical or conservative treatment (Exercise therapy, ET) in 2:1 ratio. Those randomised to ET started standardised physiotherapy within 2 weeks of the baseline appointment, while those allocated to surgery were scheduled for surgery aimed to be performed within 12 weeks of this first randomisation. In those allocated to surgery, a diagnostic arthroscopy was first carried out to rule out a rotator cuff tendon tear and other obvious intra-articular pathology requiring surgical treatment. If a rotator cuff tear large enough to require repair according to current clinical practice guidelines [12] was found, patients were excluded and the tear was repaired. Patients with a partial tear not requiring repair were included in the study. If the eligibility of the patient was confirmed in diagnostic arthroscopy, the surgeon asked a research nurse to carry out the second randomisation by opening an envelope containing the study-group assignment (Arthroscopic subacromial decompression, ASD or Diagnostic arthroscopy, DA; ratio 1:1). Only the orthopaedic surgeon and other staff in the operating room were made aware of the surgical group assignment, and they did not participate in further treatment or follow-up of the patient.

Randomisation was carried out using sequentially numbered sealed opaque envelopes. Separate randomisation lists for each three centres, with blocks varying randomly in size, were prepared by a statistician with no involvement in the clinical care of participants in the trial.

### Study interventions

#### Exercise therapy (ET)

Supervised, progressive, individually-designed physiotherapy was started within 2 weeks of randomisation using a standardised protocol that relied primarily on daily home exercises as well as 15 visits to an independent physiotherapist [13]. The details of the ET protocol are available elsewhere [10].

#### Diagnostic arthroscopy (DA)

**Fig. 1:**
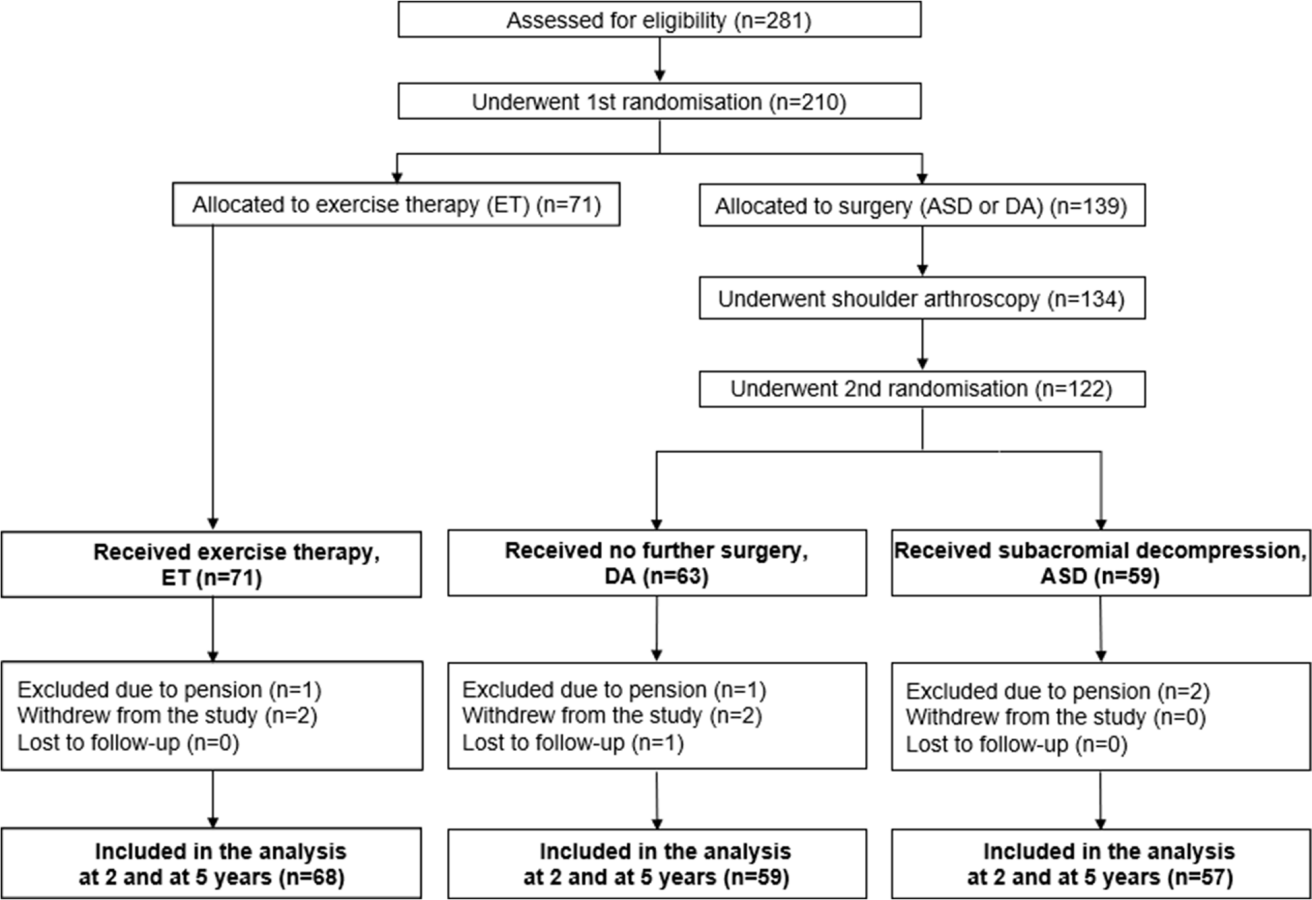
Flowchart of the Full Analysis Set

Arthroscopic examination of the glenohumeral joint and subacromial space was performed with the patient under general anaesthesia. We performed an intra-articular and subacromial assessment of the rotator cuff integrity. If arthroscopic examination revealed any pathology requiring intervention other than ASD, the patient was excluded from the trial (Fig. 1). Once the eligibility was confirmed, the participants were randomly assigned to either ASD or DA. For those allocated to the DA group, the operation was terminated. To ensure concealment of the participants and the staff other than those in the operating theatre, the DA participants were kept in the operating theatre for the time required to perform subacromial decompression [5].

#### Arthroscopic subacromial decompression (ASD)

After arthroscopic examination of the shoulder (i.e., diagnostic arthroscopy), the ASD procedure that involved the debridement of the entire subacromial bursa (bursectomy) and resection of the bony spurs and the projecting anterolateral undersurface of the acromion, was carried out with a shaver, burr and/or electrocoagulation [14].

### Postoperative care

In the ASD and DA groups, the postoperative rehabilitation was identical, consisting of one visit to an independent physiotherapist, blind to the group assignment, for guidance and instructions for home exercises.

### Outcome measures

Questionnaires were administered at baseline and 3, 6, 12, 24, and 60 months after randomisation. Work status was inquired with the following answering four options: 1) At work; 2) On sickness absence; 3) Unemployed; 4) Retired. Work status at each time point was dichotomised (At work / Not at work). The dichotomous RTW (Yes/No) was defined as follows: the study participant was at work at the primary 24-month time point or was at work at least at two of the three earlier time points. We also calculated the trajectories of return to work using dichotomous data (At work/Not at work) per each follow-up time point.

### Statistical analysis and sample size calculation

We originally powered the study to detect a difference of at least the minimal clinically important difference (15 points [15]) in the two primary outcomes, pain visual analogue scale (0–100 with 0 denoting no pain and 100 denoting extreme pain) at rest and with arm activity between the ASD and DA groups. For the study to have 90% power to show a minimal clinically important advantage of ASD over DA, under the assumption of a two-sided type 1 error rate of 5%, we planned to recruit 70 patients per group.

The present study was primarily designed to ascertain whether ASD is superior to DA in RTW with the two outcomes (the primary comparison). We also included a pragmatic comparison of the relative benefits of ASD vs. ET (the secondary comparison).

We primarily quantified the treatment effect on full analysis set basis as the difference between the groups in RTW rates up to 24 months and in work status at 24 and 60 months after the primary randomisation. In these analyses, the participants were included as randomized and we analysed all available data, the full analysis set. As a sensitivity analysis, we performed on-treatment analysis of unblinding, in which the participants were included as treated in case the participants in the DA and ET groups had received ASD after unblinding.

Categorical variables were analysed using Chi-square test and logistic regression analysis. We compared the rates of RTW between the study groups with Chi-square test. In the logistic regression model RTW was the independent variable and dependent variables were baseline work status (Yes/No), age (dichotomised by median 50.6 years), gender (Male/Female), physical work strain (dichotomised) and study group (three): Odds ratios with 95% confidence intervals and *P*-values were calculated. We estimated the prediction model accuracy by calculating a confusion matrix (actual vs. predicted) and calculated the positive and negative predictive values thereof.

All analyses are secondary by nature and must be interpreted as exploratory and supportive only. A *p* value of .05 was considered to indicate statistical significance. The estimation of the logistic regression model parameters was conducted using R 3.5.2 statistical software.

## Results

### Characteristics of the patients

There were 281 eligible patients, of whom 71 were excluded (Fig. 1). Two hundred ten patients underwent the 1st randomisation; 71 were assigned to ET and 139 to surgery. Of the 139 patients allocated to surgery, another 17 were excluded before the 2nd randomisation (Fig. 1), leaving 59 patients to receive ASD and 63 to receive DA [5].

All baseline characteristics were well balanced among the three study groups (Table 2).

**Table 2:** Patient characteristics at baseline

Characteristics	Arthroscopic subacromial decompression (***n*** = 59)	Diagnostic arthroscopy (***n*** = 63)	Exercise therapy (***n*** = 71)
Age, years	50.5 (7.3)	50.8 (7.6)	50.4 (6.6)
Female, n (%)	42 (71)	46 (73)	47 (66)
Dominant hand affected, n (%)	35 (59)	36 (57)	46 (65)
Duration of symptoms, months	18 (14)	18 (19)	22 (23)
Able to work normally regardless of shoulder symptoms, n (%)	27 (46)	31 (49)	35 (49)
Visual analogue scale score, at rest^a^	41.3 (25.8)	41.6 (25.5)	41.7 (27.5)
Visual analogue scale score, on arm activity^a^	71.2 (23.6)	72.3 (21.7)	72.4 (20.8)
Constant-Murley score^b^	32.2 (15.8)	31.7 (14.0)	35.2 (16.2)
Simple shoulder test score^c^	4.9 (2.9)	4.9 (2.9)	4.8 (2.7)
15D score^d^	0.89 (0.06)	0.89 (0.07)	0.88 (0.08)
SF-36 score physical health^e^	74.3 (12.5)	74.1 (13.1)	75.7 (10.1)
SF-36 score mental health^e^	79.4 (14.2)	77.9 (16.7)	75.6 (18.2)

Data are presented as mean (SD) unless otherwise indicated

^a^Shoulder pain at rest and at activity was assessed on a 100 mm visual analogue scale of 0 to 100, with 0 denoting no pain and 100 denoting extreme pain

^b^Scoring system for evaluation of various shoulder disorders consisting of both objective (range of motion and strength) and subjective measurements (pain assessment, workload, and leisure time activities), summarised in a score between 0 and 100; higher score indicates better shoulder function

^c^Based on 12 questions with yes (1) or no (0) response options; maximum score is 12, indicating normal shoulder function; minimum score of 0 points indicates severely diminished shoulder function

^d^Generic health related quality of life instrument comprising 15 dimensions; maximum score is 1 (full health), and minimum score is 0 (death)

^e^Generic health related quality of life instrument to quantify the physical, functional, and psychological aspects of health-related quality of life. It consists of 36 questions in eight subscales that assess physical, functional, social, and psychological well-being. Score ranges from 0 to 100, where a higher score is associated with better health

Three subjects had received a pension at the time of baseline assessment, one in each study group. Over the course of the 24-month follow-up, two patients in the ET group and two patients in the DA group withdrew from the study and one patient in the DA group was deceased, while one patient had retired before the 3-month follow-up time point in the ASD group. They all were excluded from the RTW analyses. Altogether, we had RTW data available of 184 patients (95%) during the 24-month follow-up and of 170 patients (88%) at 60 months out of the 193 randomised (Fig. 1). Another 14 patients were lost to follow-up between the 24 and 60 time points.

### Return to work outcomes

At the primary 24-month time point, 147 patients (80%) were at work, 23 (13%) on sickness absence, 10 (5%) had retired, two (1%) were unemployed or students, and data of two patients (1%) was missing. The respective figures at 60 months were 124 (67%), 26 (14%), 2 (1%), and 14 (8%). Work status at the 24-month and 60-month time points by group on the full analysis set basis are shown in Table 3. In short, there were no differences between the treatment groups in the proportion of patients at work at 24 months (Chi-square *p* = 0.84) or at 60 months (Chi-square *p* = 0.94).

**Table 3:** Work status at 24 and at 60 months by treatment group. N denotes number of patients and % percentage within group

Treatment group	ASD		DA		ET	
	N	%	N	%	N	%
**Work status at 24 months**						
**At work**	**47**	**82%**	**47**	**80%**	**53**	**78%**
Sickness absence	6	11%	8	14%	9	13%
Unemployed/student	1	2%	1	2%	0	0%
Retired	3	5%	2	3%	5	7%
Death/Withdrawn/Loss-to-F-up	0	0%	1	2%	1	1%
**TOTAL**	**57**	**100%**	**59**	**100%**	**68**	**100%**
**Work status at 60 months**						
**At work**	**38**	**67%**	**41**	**69%**	**45**	**66%**
Sickness absence	11	19%	8	14%	7	10%
Unemployed/student	0	0%	1	2%	1	1%
Retired	4	7%	5	8%	9	13%
Death/Withdrawn/Loss-to-F-up	4	7%	4	7%	6	9%
**TOTAL**	**57**	**100%**	**59**	**100%**	**68**	**100%**

**Fig. 2:**
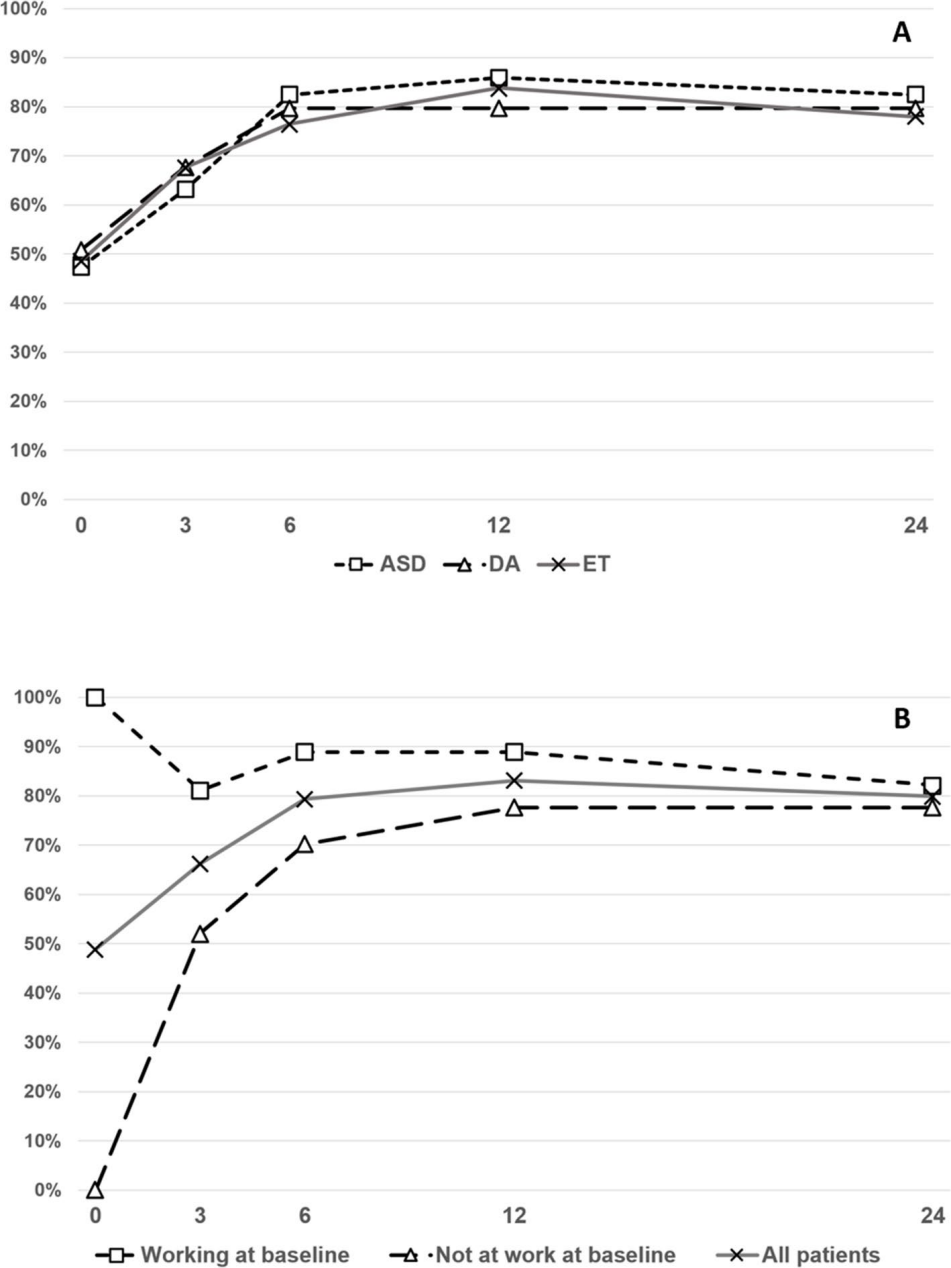
Trajectories of RTW. The Y-axis shows the proportion of patients at work (%) at each follow-up time point. The scale of X-axis is months. **A** Trajectories by treatment group: ASD denotes arthroscopic subacromial decompression, DA diagnostic arthroscopy, and ET exercise therapy. **B** Trajectories by baseline work status

Trajectories of RTW were virtually identical by time points between the study groups (Fig. 2A). Trajectories of RTW by baseline work status are shown in Fig. 2B. No difference between the three study groups was found in RTW during the 24-month follow-up period (Chi-square *p* = 0.93). In the ASD group 50 patients out of 57 (88%) returned to work at least at two time points, while the respective figures were 52 out of 59 (88%) in the DA group and 61 out of 68 (90%) in the ET group.

### Predictors of RTW

Participation in working life (odds ratio 3.48; 95% confidence interval 1.05 to 11.55) and age below the median (0.92; 0.85 to 0.99) at baseline predicted RTW in the multivariable logistic regression (Table 4). Accuracy of the prediction was 64% (117/183) and total R-square was 8%. The predictive value of the negative prediction (not returning to work) was 0.19 only, while the corresponding value of positive prediction (returning to work) was 0.94.

**Table 4:** Predictors of return to work in multivariable logistic regression: Odds ratios (OR) and 95% confidence intervals (CI)

Predictor	OR	95% CI	P-value
Study group: Diagnostic arthroscopy	1.13	0.35 to 3.69	0.84
Study group: Exercise therapy	1.32	0.42 to 4.17	0.64
Baseline work status: Working	3.48	1.05 to 11.55	0.04
Physical strain at work: Yes	0.62	0.20 to 1.93	0.41
Gender: Male	0.57	0.21 to 1.58	0.28
Age (Centred)	0.92	0.85 to 0.99	0.02

### On-treatment analysis of unblinding

After surgery, the incidence of persisting symptoms sufficiently severe to lead to unblinding of the study-group assignment was similar in the two surgically treated groups (6/59 in the ASD group and 9/63 in the DA group, *p* = 0.49). In the ASD group, 2 participants underwent a consequent re-operation, manipulation under anaesthesia for one participant while the other first had acromioclavicular resection and then later manipulation under anaesthesia. In the DA group, there were seven ASDs and one ASD coupled with subscapularis tendon repair. In the ET group, the overall incidence of surgery due to persistent symptoms was 15/71. Of these, 14 participants underwent ASD, and one underwent acromioclavicular resection. Further, three participants experienced persisting symptoms after surgery and underwent consequent reoperations.

Of the 29 patients who were unblinded, 24 (83%) returned to work while the respective figure for those who remained in their assigned study groups were 139 out of 155 (90%) (Chi-square 1.16, *p* = 0.282).

## Discussion

### Principal findings

This multicentre, randomised, placebo-controlled surgical trial involving patients with shoulder impingement syndrome shows that arthroscopic subacromial decompression (ASD) was not superior to diagnostic arthroscopy (DA) or exercise therapy (ET) in enabling patients to return to work over the course of up to 60-month follow–up. Participation in working life at baseline and younger age predicted sustainable return to work, but differences in participation in working life by these predictors were minimal at 24-month follow-up and the accuracy of the predictive model was unacceptably poor for clinical practice.

### Comparison with other studies

There are three previous studies that have reported on RTW after treatment of subacromial pain syndrome, all unblinded, open trials comparing surgery to exercise therapy [16–18]. The short- to medium-term (3 month and 2-year follow-up) results showed that there was no difference in proportion of shoulder-related absence from work between the groups [16, 17]. These findings mirror closely with the results of our ASD vs. ET comparison (Fig. 2). Two of these trials have also compared the RTW outcomes between ASD and exercise therapy at a longer, 5-year follow-up, finding no differences in self-reported work status or working ability [17, 19]. In an extended 10-year follow-up of the latter trial, there was no difference in return to work, sick leave days nor retirement rates due to shoulder problems between the groups [18].

The existing literature concerning predictors of RTW among patients with subacromial pain syndrome is very limited. Engebretsen at al [20] found that low education, probably acting as a proxy for work demands, was the most consistent predictor of pain and disability, and work status at 1-year follow-up. Also, the baseline level of shoulder symptoms, previous shoulder pain and self-reported health status predicted outcome.

### Strengths and limitations of study

As surgery has been shown to produce a profound placebo response [21–23], the actual treatment effect is impossible to distinguish from the nonspecific (and placebo) effects – such as the patients’ or researchers’ expectations of benefit – without a placebo comparison group [24]. This factor known as performance bias is a particularly problematic in trials comparing surgery to non-operative care. In our trial, it concerns primarily the ASD vs. ET comparison while the ASD vs. DA comparison was theoretically less affected, especially as similar proportion of patients in the ASD and DA groups guessed correctly whether they had undergone the actual surgery or a placebo procedure. We are also pleased with our high participation rate during the 5-year follow-up.

Our study had some limitations. The study sample calculations for the trial were based on continuous variables and the present study is apparently underpowered to detect subtle differences in the dichotomous RTW outcome variables between the treatment arms. Moreover, the number of participants with RTW data available who completed the entire 2-year follow-up was 57 and 59 in the ASD and DA groups, below the pre-specified target of 68. However, the *lack of any difference* between the treatment groups in RTW leads us to conclude that our findings are not based on insufficient evidence, as in an underpowered study, but rather on evidence demonstrating the lack of a clinically relevant treatment benefit.

Our RTW outcomes were based on self-reports by questionnaires, which may be considered as a limitation. What mitigates this concern is having a study nurse checked the work status records at each follow-up visit, looking for inconsistencies, which likely improved the quality of the data. Also, sustainable RTW is typically defined as a stable full-time or part-time RTW to either original job or a modified job for a pre-defined period of, for example, at least 3 months, without reoccurring sickness absence [25]. Our arbitrary definition of RTW consisting of being at work at the follow-up time points deviates from the common definitions for sustainable RTW, but we also assessed the trajectories of return to work per follow-up time point per group: either way, no difference between the study groups was found. Moreover, the potential bias does not differ between the AD and DA study groups since the outcome assessors were blinded to the type of operation.

In addition to our primary sham-surgery controlled efficacy comparison between ASD and DA, our study also included a pragmatic, exploratory secondary comparison between surgical and non-operative care (ASD vs. ET). In apparent contrast to four previous, similar randomised trials that found no benefit of ASD over various exercise therapy regimens [8, 9, 13, 26], we observed a small but statistically significant benefit of ASD over ET in patient-rated outcomes on pain and function at 2 years [5]. However, ASD provided no benefit over DA or ET at 5 years in the FIMPACT trial [11] and the rates of sustainable RTW did not differ between the ET and ASD study groups.

The absence of a clinically relevant benefit of ASD in the 2- and 5-year follow-up reports of this trial [5, 11] has prompted assertions that our trial design is fraught with certain methodological flaws. Allegedly, our recruited patients were too young to have shoulder impingement syndrome or were recruited too early, in so called reactive phase of the disease (< 6 months from the onset of symptoms). At baseline, the mean age of our patients was 50 years. The median duration of their symptoms was 13 months and only nine patients had had symptoms shorter than 6 months. Also, in our pre-specified subgroup analysis comparing those with symptoms less than 12 months to those with symptoms longer than 12 months, no between-group difference was found between DA and ASD groups [5]. Concerns have also been raised that a marginal resection of subacromial bursal tissue that was carried out in 30% of the patients randomised to diagnostic arthroscopy skewed our results. Our pre-specified post-hoc analysis on the potential effect of bursal tissue resection on our findings failed to show any statistically significant differences in the primary outcomes between those who had resection carried out versus those who did not. Although underpowered, the marginal differences observed in this analysis favoured no resection [5]. Another frequent assertion is that there is possibly a specific subgroup of patients who clearly benefit from the ASD procedure. This hypothesis may well be true, but it has to be tested in a randomised (placebo-surgery) controlled study. Finally, even if some “suboptimal” patients were included in our trial, they should have been equally distributed within the three randomisation groups, and accordingly, we argue that the between-group comparisons remain valid.

## Conclusions and implications

The results of this randomised controlled trial indicate that arthroscopic subacromial decompression provides no benefit over diagnostic arthroscopy or exercise therapy on return to work in patients with shoulder impingement syndrome. We also failed to find predictors for return to work that would be applicable to clinical practice. Along with previous controlled trials on the subject, these findings form a credible body of evidence that refutes the notion that patients suffering from subacromial pain syndrome will return to work faster if they have surgery. Given this body of evidence, patients should be made fully aware of the potential benefits and harms related to the various treatment options for subacromial pain and be invited to agree on a treatment plan as a fully informed participants in the shared decision-making between clinician and patient. At a policy level, we strongly encourage decision makers to consider alternatives to subacromial decompression for managing shoulder impingement symptoms.

## Data Availability

The datasets generated and/or analysed during the current study are not publicly available because the informed consent of the FIMPACT trial did not include a provision for data sharing (trial launched in 2005 when such policy was not endorsed). At present, the dataset cannot thus be shared due to a potential breach of the Finnish Personal Data Act. We intend to rectify this situation by renewing the informed consents of the trial. The corresponding author can be contacted for information related to the study.

## Abbreviations

ASD: arthroscopic subacromial decompression
DA: diagnostic arthroscopy
ET: exercise therapy
FIMPACT: Finnish Subacromial Impingement Arthroscopy Controlled Trial
RTW: return to work
